# PTEN S-nitrosylation by NOS1 inhibits autophagy in NPC cells

**DOI:** 10.1038/s41419-019-1542-0

**Published:** 2019-04-05

**Authors:** Lingqun Zhu, Chun Zhang, Qiuzhen Liu

**Affiliations:** 1 Department of Radiotherapy, Dongguan People’s Hospital, Southern Medical University, Dongguan, Guangdong China; 2 Cancer Research Institute, Southern Medical University, 1838 Guangzhou road north, Guangzhou, Guangdong China

Nitric oxide synthases are ubiquitous enzymes in malignant tumors, and known to exert both pro-tumor and anti-tumor effects. Endogenous NO is formed by three NOS isoforms (NOS1, NOS2 and NOS3) that differ in the way they modify transcription processes and enzyme activity^1^. it is a ubiquitous messenger molecule capable of regulating multiple cellular signaling pathways^2^,including those which modulate autophagy^3^. Protein S-nitrosylation is a covalent post-translational modification that results from coupling a nitric oxide (NO) moiety containing a reactive thiol group to a protein cysteine residue to form an S-nitrosothiol (SNO) moiety^4^. S-nitrosylation plays a key role in the transmission of NO-based cellular signals involved in vital cellular processes, including transcription regulation, DNA repair, apoptosis, and autophagy^5^.

Autophagy is a tightly-regulated catabolic process of cellular self-digestion by which cellular components are targeted to lysosomes for their degradation. Its functions are to provide energy and metabolic precursors under conditions of starvation and to alleviate stress by removal of damaged proteins and organelles, which is required for tumor cell survival during periods of starvation and ongoing tumorigenesis^6^. However, excessive autophagy is commonly associated with cell death^7^, and thus autophagy must be carefully regulated if cells are to survive under stressful conditions. Targeting the pro-death and pro-survival functions of autophagy has become a novel therapeutic strategy for treating cancer^8^.

Researches have reported that exogenous NO produced by the NO donor compound DETA-NONOate or the overexpression of three NOS family members creates a decrease in autophagic flux^3^. Moreover, exogenous NO can induce S-nitrosylation of JNK1 and IKKB proteins, and thereby regulate autophagy via mTOR-dependent mechanisms (IKKB-AMPK-TSC2-mTORC1) and independent mechanisms (JNK-BCL-2-Beclin1)^3^. As inhibiting the function of endogenous NOSs by L-NAME-enhanced autophagy does not require the two pathways mentioned above, endogenous NOSs might exert their effects on regulating autophagy by other mechanisms.

It is well known that mTOR is a critical regulator of autophagy^9^. However, the mTOR signaling pathway is regulated by numerous other upstream signaling pathways including PI3K/Akt. PTEN, a dual protein/lipid phosphatase, is a key regulator of the AKT/mTOR pathway. A mutation in the *PTEN* gene or a downregulation of PTEN protein production are frequent occurrences in several types of cancer, and lead to activation of the AKT/mTOR signaling pathway, which is associated with a poor clinical prognosis^10^. PTEN in neuron cells was reported to become selectively S-nitrosylated at cysteine residue (Cys-83) in the presence of low concentrations of NO^11^. Low concentrations of either exogenous or endogenous NO can selectively induce S-nitrosylation of PTEN at a specific cysteine residue (Cys-83)^12^. Moreover, S-nitrosylation of PTEN leads to degradation of PTEN via a ubiquitin ligase NEDD4-1-mediated mechanism involving ubiquitin. This degradation is followed by hyper-activation of the Akt cascade in neuron cells^13^. NOS1 is constitutively expressed in cells, and both produce low levels of NO in response to stimulation of intracellular cellular calcium flux, and stimulates various oncogenic signaling pathways such as AKT, ERK, and HIF. Thus, the existing evidence suggests that NOS1 function in promoting tumor development. However, it is unclear whether endogenous NOS1 plays a role in inducing S-nitrosylation of PTEN to form SNO-PTEN. Moreover, it remains unknown whether NOS1 play different roles in regulating autophagy, and which mechanism does it take to regulate autophagy is not clear.

In a recent article published in Cell Death Discovery, Zhu et al.^14^ have depicted the effect of exogenous NO and endogenous NO on autophagy, and also revealed the molecular mechanism of endogenous NO formed by NOS1 regulate autophagy in cancer cells. We report that NOS1 reduces excessive levels of autophagy and promotes the survival of nasopharyngeal carcinoma (NPC) cells. We found that inhibition of NOS1 increased cell death resulting from siRNA or the use of pharmacologic agents; and this effect was reversed by the autophagy inhibitor, chloroquine (CQ). The role of NOS1 in the autophagy process depended on the activation of AKT/mTOR signaling by S-nitrosylation of phosphatase and tensin homolog (PTEN) proteins (Fig. 1). The mechanism by which NOS1 modifies PTEN protein might involve a direct interaction between these two molecules. Moreover, in an *in vivo* study, the NOS1 inhibitor N(G)-nitro-L-arginine methyl ester (L-NAME) activated AKT/mTOR signaling and promoted autophagy in xenograph tumors.

**Fig. 1:**
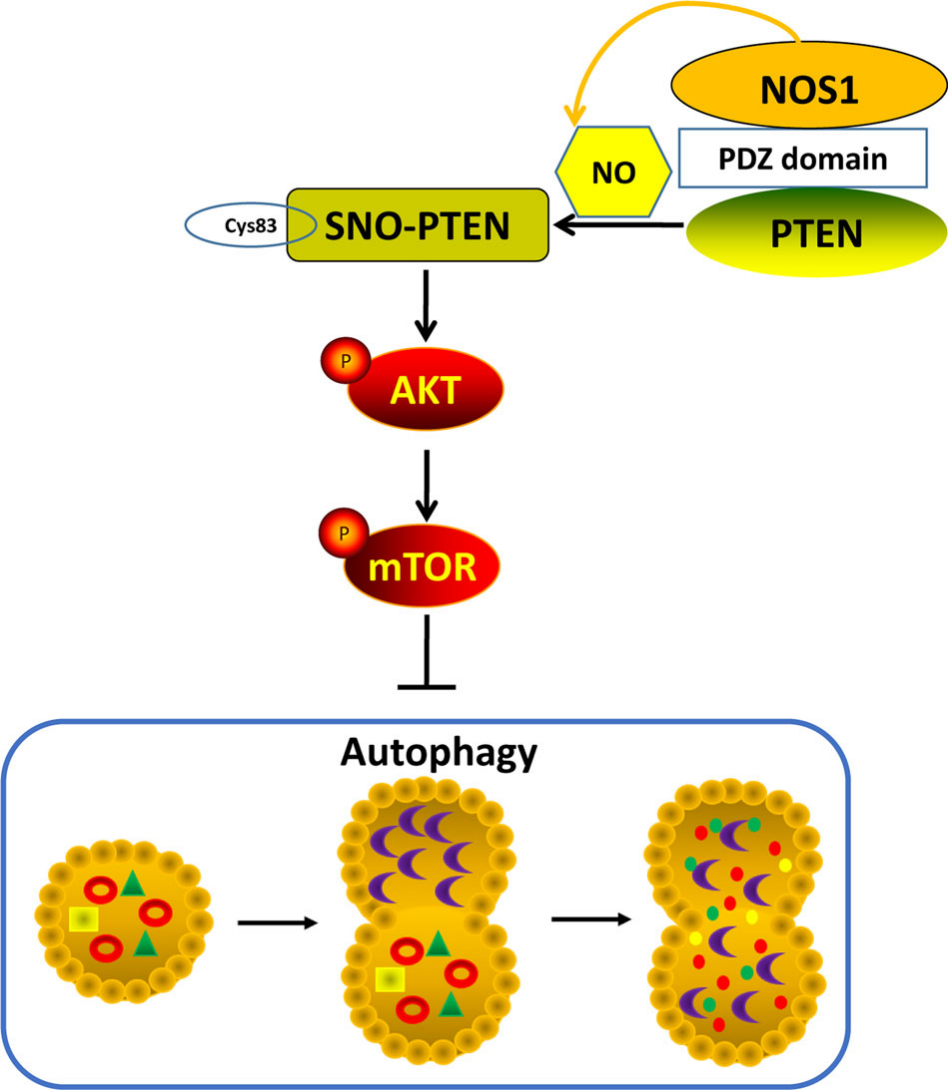
NOS1 S-nitrosylates PTEN and inhibits autophagy through activating AKT/mTOR pathway in nasopharyngeal carcinoma cells. A small amount of NO produced by NOS1 selectively modifies PTEN by S-nitrosylation, which is dependent on its interaction with PTEN. This led to activation of the AKT/mTOR signaling pathway, the down-regulation of autophagy

Although all three NOSs (NOS1, NOS2, and NOS3) exist in NPC cells and contribute to the total NO concentration in NPC cells, only NOS1 was shown to activate AKT/mTOR signaling by S-nitrosylation of PTEN. NOS2 produced higher levels of NO in CNE2 cells, it only slightly S-nitrosylated PTEN. NO has a very short biological half-life^2^. One possible factor underlying the special ability of NOS1 to nitrosylate PTEN might be the unique PDZ domain possessed by NOS1^11^. A PDZ domain consists of ~ 90 amino acids. Modular interactions mediated by PDZ domains facilitate the selective and effective interaction of NOS1 with its target substrate. In brain tissue, NOS1 is targeted towards synaptic membranes by its interactions with scaffolding proteins PSD-95 and PSD93, which anchor two PDZ domains^11^.NOS1 interacts with its substrate by recognizing and selectively binding the specific C-terminus of its target protein molecule via the PDZ domain. PTEN possesses a C-terminal PDZ-binding motif (PDZ-BM) that is recognized by a specific set of PDZ domains found in scaffolding and regulatory proteins. Many of the proteins that interact with PTEN via PDZ domains are multi-PDZ-domain scaffolding proteins that stabilize PTEN and decrease p-AKT levels^15^. However, the binding of PTEN to a specific PDZ domain containing NOS1 has not yet been reported. For the first time, this study verified that NOS1 selectively modifies PTEN by S-nitrosylation, which is dependent on its interaction with PTEN. Future studies will verify whether NOS1 directly or indirectly interacts with PTEN via scaffolding and regulatory proteins containing PDZ domains.

In conclution, Zhu et al. current findings help to elucidate the effects of NOS1 on autophagy, and provide a detailed molecular mechanism explaining the efficacy of NOS1 in modulating excessive autophagy via S-nitrosylation of PTEN. The affects produced by NOS1 contribute to cell survival and impact the development of chemoresistance. As a result, these findings may provide clues for improving the treatment of nasopharyngeal carcinoma. The capacity of these compounds to provide a means of cancer cell death that enhances the effects of standard therapies should be taken into consideration for designing novel therapeutic strategies. Moreover, and alternatively, the regulation of autophagy by NOS1 if controlled should be considered as a potential therapeutic strategy for cancer.

## References

[1] Xu W (2002). Cell Res..

[2] Thomas DD (2008). Free Radic. Biol. Med..

[3] Sarkar S (2011). Mol. Cell.

[4] Wang Z (2012). Cancer Lett..

[5] Haldar SM (2011). Mol. Cell.

[6] Kroemer G (2010). Mol. Cell.

[7] Notte A (2011). Biochem. Pharmacol..

[8] Fulda S (2015). Oncogene.

[9] Yang Z (2010). Curr. Opin. Cell Biol..

[10] Yuan TL (2008). Oncogene.

[11] Hillier BJ (1999). Science.

[12] Numajiri N (2011). Proc. Natl. Acad. Sci. USA.

[13] Kwak YD (2010). Mol. Neurodegener..

[14] Zhu L (2017). Cell Death Discov..

[15] Sugi T (2008). Biochem. Biophys. Res. Commun..

